# Swallowing Artificial Teeth

**Published:** 1882-12

**Authors:** 


### SWALLOWING ARTIFICIAL TEETH.

The *Lancet* reports the swallowing of a set of artificial teeth by a man in an epileptic fit. The denture was expelled per anum within twenty-four hours. After cleaning the plate, the patient placed it in situ, and all was well for nine years, when it again descended and was thirty-one days in passing out.

